# Molecular doping of nucleic acids into light emitting crystals driven by multisite-intermolecular interaction

**DOI:** 10.1038/s41467-022-33999-y

**Published:** 2022-10-19

**Authors:** Woo Hyuk Jung, Jin Hyuk Park, Seokho Kim, Chunzhi Cui, Dong June Ahn

**Affiliations:** 1 Department of Chemical and Biological Engineering, Korea University, Seoul, 02841 Korea; 2 KU-KIST Graduate School of Converging Science and Technology, Korea University, Seoul, 02841 Korea; 3 Department of Chemistry, National Demonstration Centre for Experimental Chemistry Education, Yanbian University, Yanji, 133002 China

**Keywords:** Bioinspired materials, DNA and RNA

## Abstract

We reveal the fundamental understanding of molecular doping of DNAs into organic semiconducting tris (8-hydroxyquinoline) aluminum (Alq_3_) crystals by varying types and numbers of purines and pyrimidines constituting DNA. Electrostatic, hydrogen bonding, and π-π stacking interactions between Alq_3_ and DNAs are the major factors affecting the molecular doping. Longer DNAs induce a higher degree of doping due to electrostatic interactions between phosphate backbone and Alq_3_. Among four bases, single thymine bases induce the multisite interactions of π-π stacking and hydrogen bonding with single Alq_3_, occurring within a probability of 4.37%. In contrast, single adenine bases form multisite interactions, within lower probability (1.93%), with two-neighboring Alq_3_. These multisite interactions facilitate the molecular doping into Alq_3_ particles compared to cytosines or guanines only forming π-π stacking. Thus, photoluminescence and optical waveguide phenomena of crystals were successfully tailored. This discovery should deepen our fundamental understanding of incorporating DNAs into organic semiconducting crystals.

## Abstract (web summary)

DNAs exhibit a unique molecular doping structure, unseen in conventional hybrid assemblies, when doped into light-emitting organic materials. Here authors unveil doping mechanism of DNAs into organic semiconducting crystals and tailor their photoluminescence and optical waveguide phenomena.

## Introduction

Since their inception as a typical genetic information carrier, nucleic acids have become a member of the material field and are widely used^1–4^. The unique physical and chemical properties make nucleic acid-associated materials the focus of numerous studies. For example, a nucleic acid molecule is generally complexed with π-conjugated organic semiconductors and serves as (i) an efficient receptor element for recognizing biological/chemical targets^5–7^, (ii) a template for the assembly and polymerization of organic semiconductors^8–10^, (iii) a walking component in a light-driven artificial nanomachine^11,12^, (iv) a wide-bandgap material in organic light-emitting diodes enhancing their luminescence efficiency^2,13,14^, (v) a molecular gadget for tuning organic semiconductor crystals bio-active when properly hybridized^15^, and (vi) a biological moiety of organic hybrid crystals for remote sensing via optical waveguide effects^16^.

Hybrid assemblies have become important in the field of self-assembly^17,18^. Binary or ternary hybrid assemblies have been prepared through molecular doping between organic semiconducting components^19^, involving noncovalent intermolecular interactions, such as van der Waals force, π-π stacking, and hydrogen bonding^20–22^. The forms of hybrid assemblies can be classified into hetero structures^23,24^ and uniform-^25,26^ or gradient-doped^27^ structures. However, deoxyribonucleic acids (DNAs) doped into light-emitting organic crystals exhibit distinctly different structures of molecular doping that has been unseen in conventional hybrid assemblies^3,15^. To date, studies have focused on the application of DNA-hybrid assemblies; however, little attention has been paid to how these nucleic acids interface with organic components at the molecular level. A fundamental understanding of the intermolecular interactions between nucleic acids and light-emitting organic molecules can lead to the rational design of these hybrid assemblies, playing a key role in the further development of biophotonics.

This study proposes an interpretation of the molecular doping of oligonucleotide DNA molecules in light-emitting crystals at the molecular level. A typical light-emitting organic material, tris (8-hydroxyquinoline) aluminum (Alq_3_), was chosen as the model molecule. Alq_3_ crystals doped with nucleic acid molecules were assembled through a simple reprecipitation method by varying the length of DNA chains and base moieties type. The intermolecular interfacing behavior between nucleic acids and Alq_3_ is interpreted in terms of electrostatic, hydrogen bonding, and π-π stacking interactions.

## Results

### Non-uniform molecular distribution of DNA in light-emitting organic particles

**Fig. 1:**
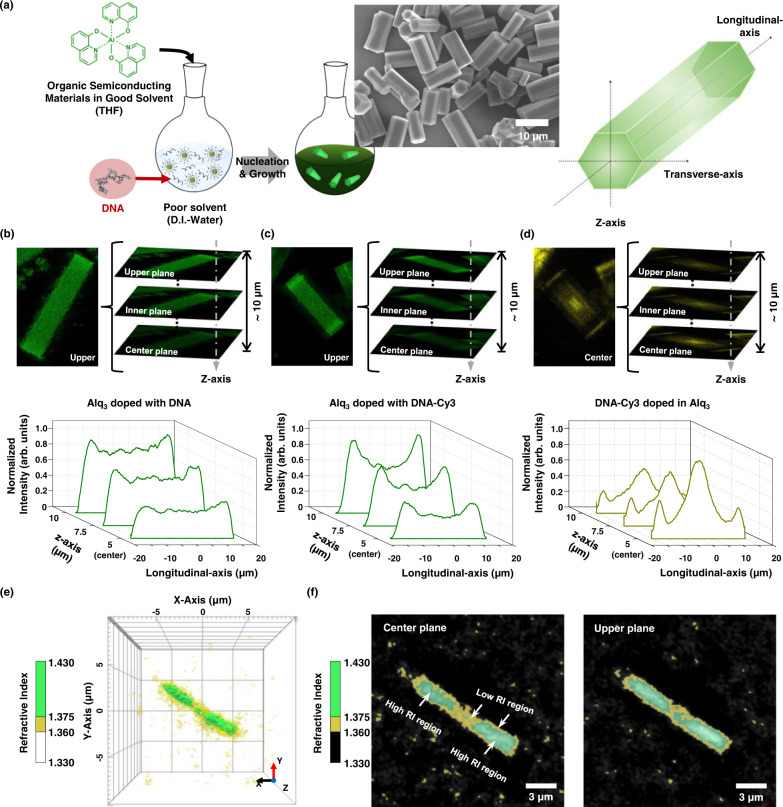
DNA as a molecular dopant in light-emitting organic crystals. **a** Schematic illustration of the fabrication of DNA-doped Alq_3_ microparticles (left) and definition of coordination of hexagonal microrods (right). Z-stack confocal laser scanning microscopy images (top) and longitudinal normalized fluorescence profiles averaged from five separate particles from the upper to the center plane (bottom) of (**b**) DNA doped Alq_3_ molecules, (**c**) DNA-Cy3 doped Alq_3_ molecules, and (**d**) DNA-Cy3 molecules in a particle. The single-stranded DNA sequence for fabricating the particles is 5′-ATC CTT ATC AAT ATT TAA CAA TAA TCC-3′. The images and profiles of Alq_3_ molecules are obtained by excitation using a 405 nm laser; a 300‒550 nm filter is used. The images of the DNA-Cy3 molecules are obtained by excitation with a 555 nm laser; a 300‒630 nm filter is used. The fluorescence of DNA-Cy3 molecules is enhanced by 3-fold for clarity. The fluorescence profiles of Alq_3_ and DNA-Cy3 molecules are normalized by the intensity of Alq_3_ from sides of particles at upper plane and the intensity of DNA-Cy3 from center of particles at center plane, respectively. **e** 3D refractive index (RI) distribution of the DNA-doped Alq_3_ particle. The low RI regions ranging from 1.360 to 1.375 and high RI regions ranging from 1.375 to 1.430 are represented by yellow and green surfaces, respectively. **f** Cross-sectional images of the corresponding 3D RI distributions on the center plane (left panel) and the upper plane (right panel) of the particle. High and low RI regions were colored as green and yellow, respectively. Low RI regions become larger at the center, especially at the center plane of the particle. Source data are provided as a Source Data file.

Figure 1a presents a schematic of the bio-functional Alq_3_ microparticles doped with DNA and illustrates the axial direction of the hexagonal particles. We used confocal laser scanning microscopy (CLSM) to quantify the internal fluorescence distribution of Alq_3_ and DNA molecules in particles. The Alq_3_ and DNA molecules, labeled with Cy3 fluorescent dye, are excited using a 405 and 555 nm laser, respectively. The main PL peaks of the Alq_3_ particles and Cy3 dyes are observed at ~512 and ~572 nm (Supplementary Fig. 1a), in agreement with the known characteristics^28^. Additionally, the PL spectrum of Alq_3_ particles overlaps with the absorption spectrum of DNA-Cy3 molecules. Thus, DNA molecules labeled with Cy3 generate Förster resonance energy transfer (FRET) effects from Alq_3_ to a fluorescent dye with a FRET efficiency of 7.9% and slightly lower the emission intensity of Alq_3_ molecules compared to Alq_3_ doped with DNA (Supplementary Fig. 1b). Examples of confocal technical data related to individual z-stack images, three-dimensional (3D) rendered images, and average profiling analysis in the longitudinal direction of Alq_3_ doped with anthrax lethal factor 27-mer DNA, Alq_3_ doped with DNA-Cy3, and DNA-Cy3 molecules are provided in Fig. 1b, c, and d, respectively. The Alq_3_ molecules are distributed into 20 μm particles, while the DNA molecules have a strikingly unique distribution in these images. The Alq_3_ molecules have higher intensity in the upper plane than in the particles’ central plane (10 μm inner plane). The fluorescence intensity of the Alq_3_ molecules is uniform along the longitudinal axis of particles doped with DNA (Fig. 1b) while that of the sides of particles is slightly higher due to the optical waveguide effects of crystals toward the edges^16,29,30^. The fluorescence intensity of the Alq_3_ molecules on the center of particles doped with DNA-Cy3 becomes lower (Fig. 1c). Thus, the difference between the fluorescence intensities on the center and sides of particles doped with DNA-Cy3 increases compared to Alq_3_ particles doped with DNA. The fluorescence of the DNA-Cy3 molecules is most intense at the center of the particles, and the central plane has a higher intensity than the upper plane (Fig. 1d). It indicates that the DNA molecules, concentrated at the center, generate FRET from Alq_3_ to the Cy3 fluorescent dye. Besides via the fluorescence distribution, we further confirmed the molecular distribution of doped-DNA molecules in Alq_3_ particles via the refractive index (RI) distribution. As the RI of the Alq_3_ is larger than the RI of the DNA^3^, the distribution of DNA molecules in the Alq_3_ particles can be visualized through the holotomographic microscopy observing the difference in RI. In the reconstructed 3D RI distributions of DNA doped Alq_3_ particle (Fig. 1e), yellow and green surfaces represent, respectively, low RI regions ranging from 1.330 to 1.375 and high RI regions ranging from 1.375 to 1.430. The yellow region distributes at the surfaces and center, compared to the rest green region, indicating that DNA molecules with a low RI exist mainly on the surfaces and in the center of the particle. We then obtained cross-sectional images of corresponding 3D RI distribution at the center and upper planes along the *z*-axis (Fig. 1f). The low RI regions in the particle (yellow-colored) exist at the center region of the particle at both planes; however, the yellow region becomes larger at the center plane, indicating that the DNA molecules are more concentrated, which is consistent with the fluorescence distribution of DNA molecules in Alq_3_ particles given in Fig. 1d. These results suggest that the DNA molecules are nonuniformly doped into the Alq_3_ particles.

### Effect of DNA length on molecular doping of DNAs

**Fig. 2:**
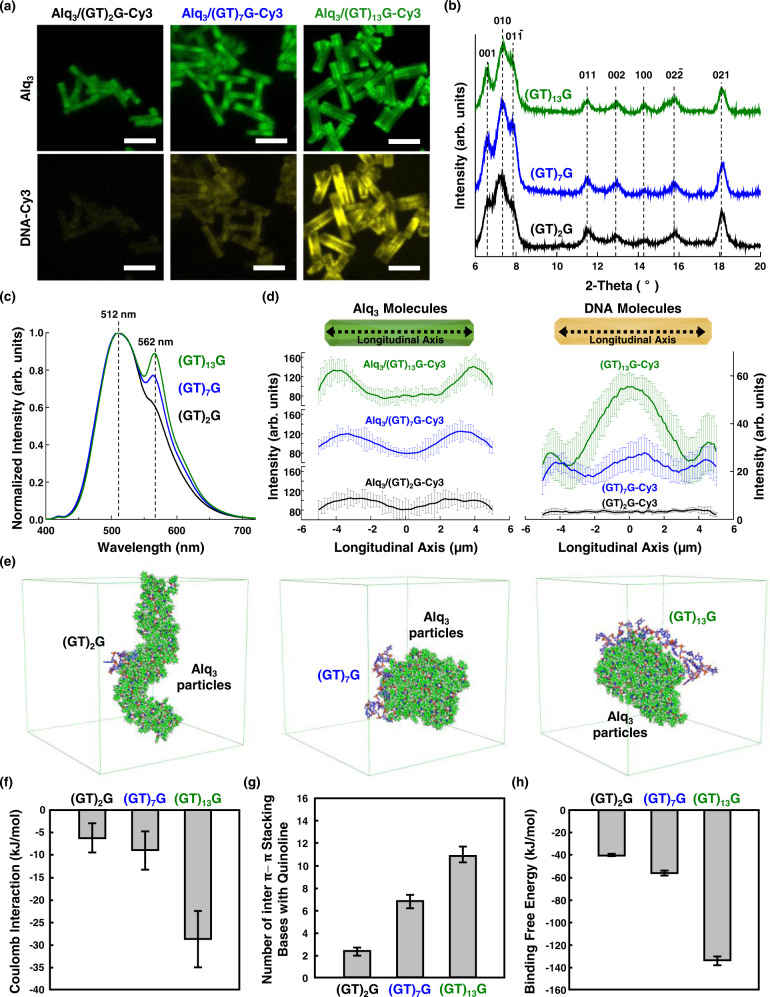
Effect of DNA length on molecular doping of DNAs into Alq_3_ microparticles. **a** CLSM images captured at the center plane of Alq_3_/DNA[(GT)_x_G]-Cy3 particles; *x* = 2, 7, and 13 (scale bar, 10 μm). **b** XRD patterns of all DNA-doped Alq_3_ particles show α−phase peak for Alq_3_ at 11.55° and 12.94°, along with a δ-phase peak at 11.79°. **c** PL spectra with excitation at 365 nm, revealing FRET properties of Alq_3_/DNA[(GT)_x_G]-Cy3 particles; *x* = 2, 7, and 13. **d** Longitudinal fluorescence profiles quantifying the local intensity of Alq_3_ (left) and DNA[(GT)_x_G]-Cy3 molecules (right) at the center plane averaged from six separate particles. CLSM images and profiles were obtained with excitation by a 405 nm laser for the Alq_3_ molecules and 555 nm laser for the DNA-Cy3 molecules used for doping. The filters used for observing the Alq_3_ and Cy3 molecules ranged from 300 to 550 nm and from 300 to 630 nm, respectively. The concentration of DNA in this experiment is 800 nM. **e** Molecular dynamics simulation for the length-dependent molecular doping of DNAs. Final configurations were shown for Alq_3_ assembly with DNA[(GT)_x_G] (*x* = 2, 7, and 13). **f** Electrostatic interaction between Alq_3_ particles and DNAs, and (**g**) averaged number of bases that form inter π-π stacking between bases of DNAs and quinoline of Alq_3_ assembly during the final 10 ns of simulations. **h** Calculated free energy change (binding free energy) of the DNAs interacting with Alq_3_ assembly, obtained from umbrella sampling. Error bars in (**d**), (**f**), (**g**), and (**h**) represent standard deviation. Source data are provided as a Source Data file.

The length of the oligonucleotides of DNAs in the Alq_3_ microparticles was controlled to observe the molecular doping of DNAs and changes in the optical features of the particles (Fig. 2). In this experiment, DNAs with (GT)_2_G (5-mer), (GT)_7_G (15-mer), and (GT)_13_G (27-mer) nucleotides were labeled with Cy3 fluorescence dye to observe their molecular distribution. The concentration of DNAs used in this experiment was 800 nM. The particle size is independent of the length of the nucleotides doped in the Alq_3_ microparticles, as determined from the CLSM (Fig. 2a) and scanning electron microscopy (SEM) images (Supplementary Fig. 2). The corresponding X-ray diffraction (XRD) patterns, in Fig. 2b, of DNA-doped Alq_3_ particles show typical Alq_3_ α-phase peaks at 11.40° and 12.81°, and δ-phase peak at 11.79°, as observed in earlier studies^31,32,33^. The normalized photoluminescence (PL) spectra of DNA-Cy3-doped Alq_3_ microparticles are shown in Fig. 2c. The main PL peaks of the Alq_3_ particles and Cy3 dyes are observed at ~512 and ~572 nm, respectively^28^; the peak of Alq_3_ corresponds to yellowish-green emission from α- and δ-phases, consistent with the observed XRD patterns. Interestingly, the emission ratio calculated by the Gaussian peak area after deconvolution (*Α*_572 nm_/*Α*_512 nm_) increased from 0.10 to 0.30, as the number of nucleotides increased from 5 to 27. Furthermore, the emission ratio (*Α*_572 nm_/*Α*_512 nm_) of Alq_3_ microparticles doped with (GT)_2_G-Cy3 (0.11) is still low after increasing the concentration of DNA-Cy3 to 1500 nM (Supplementary Fig. 3) compared to the emission ratio of Alq_3_ microparticles doped with (GT)_7_G-Cy3 and (GT)_13_G-Cy3. The efficiency of energy transfer between the Alq_3_ donor and Cy3 dye acceptor is expected to be maximal when the distance between the two is minimal^34^. However, the longest DNA molecule [(GT)_13_G)] affords the highest energy transfer efficiency among the samples tested. The insight into this unrevealed factor influencing the energy transfer between Alq_3_ and DNA in these hybrid particles was obtained from the molecular distributions determined by CLSM. The CLSM images (Fig. 2a, bottom panel) indicate that DNA-Cy3 molecule distribution differs depending on the DNA length; the yellow-fluorescence signal from the Cy3 molecules is more intense for longer DNA lengths. The average local fluorescence intensities of Alq_3_ and DNA molecules are shown in Fig. 2d. The longitudinal fluorescence profile of the DNA molecules (Fig. 2d, right) in the Alq_3_/DNA[(GT)_2_G]-Cy3 particle has low intensity across the entire region, that is, little DNA is doped into these particles. The fluorescence of DNA molecules in Alq_3_/DNA[(GT)_7_G]-Cy3 is spread across the entire region, while that in Alq_3_/DNA[(GT)_13_G]-Cy3 is most intense in the central region, indicating that longer DNAs are more strongly doped into the particles. The DNA distribution in the particles affects energy transfer from the Alq_3_ molecules. The fluorescence intensity of the Alq_3_ molecules (Fig. 2d, left) is relatively uniform for particles with the shortest DNA[(GT)_2_G]-Cy3 except that that the fluorescence intensity of the edges of particles is slightly higher due to the optical waveguide effects. By contrast, the fluorescence intensity of the Alq_3_ molecules becomes lower at the center of the particles with DNA[(GT)_7_G]-Cy3 and DNA[(GT)_13_G]-Cy3 along the longitudinal axis. These results indicate that the energy transfer from the Alq_3_ donor to the Cy3 dye acceptor is largest at the highest degree of doping.

All atomic molecular dynamics (AA MD) simulations were performed to interpret factors inducing increased doping for longer DNA segments. The final configurations of the three systems with 100 Alq_3_ molecules and DNA[(GT)_*x*_G] (*x* = 2, 7, and 13) are shown in Fig. 2e. All DNA segments are adsorbed onto the Alq_3_ particles. The electrostatic interaction (Fig. 2f) and number of π-π stacking structures (Fig. 2g) were obtained from the final 10 ns of the simulations. Longer DNA segments induce stronger electrostatic interactions between the phosphate backbone and Alq_3_ particles, facilitating the molecular doping of DNAs. In this acidic system, H^+^ ligands are present around the O cation of Al-O; thus, Alq_3_ is positively charged via the absorption of H^+^ by the 8-hydroxyquinoline moiety^35^. The dissociation constant (p*K*_a_) of the phosphodiester group of DNA is approximately 1^36^, thereby maintaining negative charges on the DNA backbone^37^. This implies that the negative charges of DNA and the positive charges of the Alq_3_ molecules can engender more intense intermolecular attraction for longer DNA segments. Moreover, larger numbers of π-π stacking structures are observed between the DNA bases and quinoline of Alq_3_ particles for longer DNA segments. We then performed a series of steered molecular dynamics (SMD) simulations to quantify the free energy change before and after adsorption of different length DNA segments, as shown in Fig. 2h. The binding free energies of DNA[(GT)_*x*_G] (*x* = 2, 7, and 13) adsorbed onto the Alq_3_ particles are −39.9, −56.8, and −134.4 kJ/mol, respectively. From these results, it is reasonable to deduce that longer DNA molecules experience stronger interaction, including electrostatic attraction and π-π stacking, with Alq_3_, thereby increasing the extent of doping.

The effect of electrostatic attraction on the molecular doping of DNAs was further investigated by assembling Alq_3_ particles with DNAs in aqueous solutions of various pH values. The particle morphologies and molecular distributions of the DNA-doped Alq_3_ particles at various pH are shown in Supplementary Fig. 4. At pH 4, 7, and 10, the particles have regular prismatic hexagonal shapes, while no defined microstructures are observed at pH 3 and 12. CLSM images captured at the z-centric planes show that the Alq_3_ (middle) and DNA-Cy3 (bottom) molecules in these particles are distributed differently at various pH (Supplementary Fig. 4). Green fluorescent microparticles were observed at pH 4, 7, and 10. Specifically, efficient doping of DNA molecules into the Alq_3_ particles is observed at pH 4, and no yellow-fluorescence signals of the DNA-Cy3 molecules are observed in particles fabricated at other pH values. The quantitative histogram of DNA-Cy3 molecules also shows that the DNAs are predominantly incorporated into the Alq_3_ particles at pH 4 (Supplementary Fig. 5a). The PL spectra of Alq_3_ microparticles doped with DNA-Cy3 at various pH values are shown in Supplementary Fig. 5b. The emission ratio calculated by the Gaussian peak area (*Α*_572 nm_/*Α*_512 nm_) is 0.23 at pH 4, indicating significant doping, and ~0.07 at other pH values. It appears that the intermolecular interaction is pH-dependent; the electrostatic attraction between positively charged Alq_3_ and DNA with a relatively strong negative charge is dominant at pH 4. This is consistent with the report that the binding between aluminum ions and calf thymus DNA is pH dependent^38^. The binding constant increased in the pH range of 3.5‒4.5, reaching a maximum at pH 4.5. However, at pH above 5.5, the binding constant declined, and no binding of aluminum ions to DNA is observed at pH 6.0 and 7.0. Therefore, the acidity of the aqueous solutions used in the assembly of the Alq_3_ particles should be controlled to generate strong electrostatic attraction, inducing the incorporation of DNA molecules.

### Effect of base units on molecular doping of DNAs

**Fig. 3:**
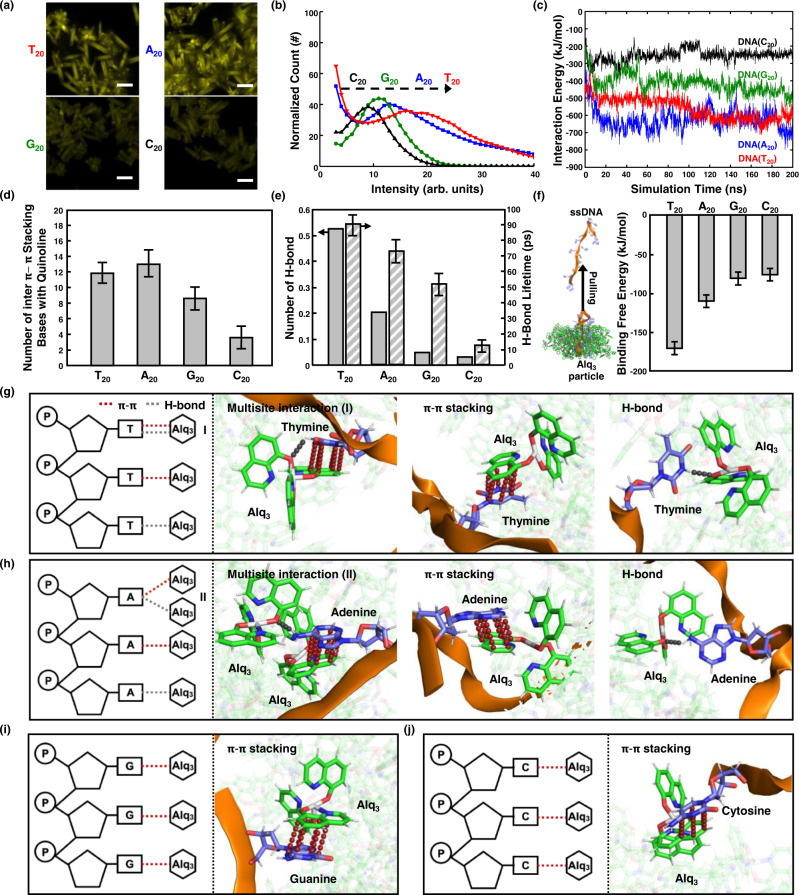
Effect of base units on molecular doping of DNAs possessing distinct intermolecular interactions. **a** Fluorescent analysis of doped DNA(T_20_, A_20_, G_20_, and C_20_)-Cy3 in Alq_3_ particles. CLSM images are obtained with excitation by a 555 nm laser (scale bar, 10 μm). **b** Quantitative histogram of doped DNA-Cy3 using CLSM. Molecular dynamics simulations for the base-dependent incorporation of DNAs into Alq_3_ microparticles. **c** Time-traced total interaction energy between Alq_3_ particles and DNAs. **d** Number of bases that form inter π-π stacking with the quinoline of Alq_3_ assembly during simulation. **e** Number of hydrogen bonds formed between Alq_3_ molecules and DNAs and their corresponding lifetimes during the final 100 ns simulation. **f** Binding free energy of the DNAs on Alq_3_ assembly, obtained from umbrella sampling for three replicates of DNAs fully adsorbed on Alq_3_ particles. The schematics and molecular views of (**g**) T_20_, (**h**) A_20_, (**i**) G_20_, and (**j**) C_20_ doped in Alq_3_ particles from the final 10 ns of AA MD simulation. Three key interactions, including multisite interaction, stoichiometric π-π stacking, and hydrogen bonding, for doping of T_20_ and A_20_ into Alq_3_ are shown in (**g**) and (**h**), respectively. π-π stacking interaction doping G_20_ and C_20_ into Alq_3_ are shown in (**i**) and (**j**), respectively. The hydrogen bonds and π-π stacking interaction in schematics and molecular views are depicted in gray and red dashed lines, respectively. Error bars in (**d**), (**e**), and (**f**) represent standard deviation. Source data are provided as a Source Data file.

To explore the molecular doping of DNA with respect to the purine and pyrimidine base units, we fabricated Alq_3_ particles with DNAs having different patterns of the same length. In this experiment, four patterns were considered: poly(T), poly(A), poly(G), and poly(C) with a 20-mer sequence. The morphological features of DNA-Alq_3_ particles are presented in Supplementary Fig. 6. The Alq_3_ molecules crystallized similarly when doped with the 20-mer DNAs. Interestingly, the fluorescence signals in the CLSM images indicate that DNA(T_20_)-Cy3 and DNA(A_20_)-Cy3 are more effectively doped into the Alq_3_ particles than the other DNAs (Fig. 3a). The normalized quantitative histogram obtained from CLSM image shows the most intense fluorescence in particles doped with DNA(T_20_)-Cy3 (Fig. 3b). The PL spectra of the samples are shown in Supplementary Fig. 7. The emission at 572 nm after excitation at 500 nm is most intense for DNA(T_20_), consistent with the quantitative histogram of CLSM images. AA MD simulations were conducted to identify the binding phenomena, inducing doping between different bases and Alq_3_ particles. We constructed systems using 100 Alq_3_ molecules and DNA segments with 20-mer sequences: poly(T), poly(A), poly(G), and poly(C). The final configurations of the four systems are shown in Supplementary Fig. 8. The DNA segments with poly(T) and poly(A) sequences are fully adsorbed onto the Alq_3_ particles, whereas poly(G) and poly(C) sequences are partially adsorbed. The interaction energy between the DNA segments and Alq_3_ particles (Fig. 3c) also indicates strong interaction of DNA(T_20_) and DNA(A_20_) with the Alq_3_ particles than the other DNAs, leading to full adsorption on the Alq_3_ particles. Furthermore, we monitored the π-π stacking interaction (Fig. 3d) and hydrogen bonding (Fig. 3e) between the Alq_3_ particles and DNA segments to clarify differences in the interaction energy of the DNA molecules. Larger numbers of π-π stacking structures and hydrogen bonds are observed for DNA(T_20_) and DNA(A_20_). The average hydrogen bond lifetimes also show that Alq_3_ forms stronger hydrogen bonds with thymine and adenine than with guanine and cytosine. A series of SMD simulations were performed to calculate the binding free energy of fully adsorbed DNAs. The initial configurations for the calculation were derived from Alq_3_ with DNA(A_20_) to ensure that the four DNA segments are fully adsorbed on the Alq_3_ particles (see details in Methods section). The binding free energies averaged over three replicates of DNAs (T_20,_ A_20,_ G_20,_ and C_20_) adsorbed on the Alq_3_ particles are ‒170.5, ‒110.4, ‒81.3, and ‒76.6 kJ/mol, respectively (Fig. 3f), indicating that DNAs (T_20_ and A_20_) with relatively large binding free energy tend to be fully adsorbed onto Alq_3_ particles (Supplementary Fig. 8). By closely inspecting how base units form noncovalent interactions, including π-π stacking and hydrogen bonding during the final 10 ns of simulations, we show key interactions that make DNA(T_20_ and A_20_) strongly doped into Alq_3_ (Fig. 3g and Table 1). Both π-π stacking and hydrogen bonds are frequently formed between the bases (thymine and adenine) and Alq_3_ molecules. In particular, 56 thymine molecules form multisite interactions with single Alq_3_ molecules via both π-π stacking and hydrogen bonding, whereas 26 adenine molecules form hydrogen bonds with one of the Alq_3_ molecules and subsequent π-π stacking interactions with neighboring Alq_3_ molecules. Considering the higher occurrence probability (4.39%) of multisite interactions and the strongest binding energy (‒170.5 kJ/mol) with Alq_3_ particles, we conclude that the multisite interaction of π-π stacking and hydrogen bonding between thymine and single Alq_3_ molecules causes the strongest attraction and intensifies the extent of doping into Alq_3_ particles. In comparison, few hydrogen bonding interactions with Alq_3_ molecules are observed for DNA(G_20_) and DNA(C_20_). Only π-π stacking interactions are involved in the doping of DNA(G_20_) (Fig. 3i and Table 1) and DNA(C_20_) (Fig. 3j and Table 1). The adsorption energies of the DNA(T_20,_ A_20,_ G_20,_ and C_20_) on the Alq_3_ crystal surface were also calculated (Supplementary Fig. 9). The adsorption energy is highest for thymine molecules on the Alq_3_ crystal surface. Combined with experimental results, we confirm that thymine molecules induce stronger attraction with Alq_3_, suggesting that the oligonucleotide constituents control molecular doping in light-emitting organic crystals.

**Table 1:** Summary of the intermolecular interactions between Alq_3_ and DNA with different bases during the final 10 ns of AA MD simulations

	Numbers for the Occurrence of Interactions (Probability, %)			
	Multisite interaction (Type I)	Multisite interaction (Type II)	H-bond	π-π stacking
**T**_**20**_	56 (4.39%)	-	81 (6.34%)	1140 (89.3%)
**A**_**20**_	-	26 (1.93%)	40 (2.96%)	1284 (95.1%)
**C**_**20**_	-	-	1 (0.20%)	491 (99.8%)
**G**_**20**_	-	-	1 (0.10%)	823 (99.9%)

### Tailored bio-specific photonics and optical waveguide effects of DNA-Alq_3_ hybrid crystals

**Fig. 4:**
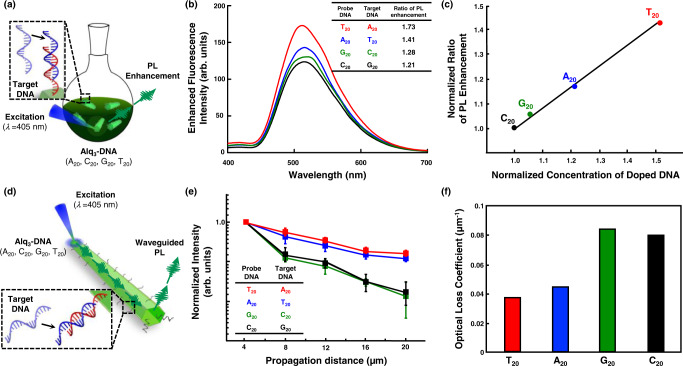
Bio-specific photoluminescence and optical waveguide phenomena of Alq_3_ microparticles upon complementary DNA-DNA hybridization. **a** Schematic illustration for observing bio-recognitive photonics of Alq_3_ doped with DNAs. **b** PL spectra of Alq_3_/DNAs (T_20_, A_20_, G_20_, and C_20_) after treatment with corresponding complementary DNAs (excitation at 365 nm). The inset chart represents the ratio of PL enhancement compared to PL intensity before complementary DNA treatment (see details in Supplementary Fig. 10). **c** Normalized ratio of PL enhancement as a function of the normalized concentration of doped DNA-Cy3 molecules. Concentration is derived from the fluorescence intensity of doped DNA-Cy3 molecules. **d** Schematic illustration for measuring optical waveguide phenomena of Alq_3_ microparticles doped with DNAs. **e** Normalized intensity of waveguided PL from Alq_3_/DNAs (T_20_, A_20_, G_20_, and C_20_) microparticles after treatment with corresponding complementary DNAs. **f** Optical loss coefficient of each Alq_3_ microparticle, calculated by exponentially fitting the emission intensities with respect to the propagation distance. Error bars in (**e**) represent standard deviation. Source data are provided as a Source Data file.

Fluorescence enhancement (Fig. 4a) and change in optical waveguide efficiency (Fig. 4d) of Alq_3_ particles with DNAs were observed after treatment with specific target DNA (tDNA) molecules to investigate the resulting biophotonics of Alq_3_ particles. The tDNAs used in this experiment were A_20_, T_20_, C_20_, and G_20_, for which the hybridization was complementary to single-stranded DNAs (T_20_, A_20_, G_20_, and C_20_), respectively. As shown in Fig. 4b, the largest fluorescence enhancement is observed when DNA(T_20_) doped in Alq_3_ particles interact with tDNA (A_20_). The relative ratio of PL enhancement is plotted as a function of the concentration of DNA-Cy3 doped into the Alq_3_ (Fig. 4c). After interacting with complementary tDNAs, the ratio increases linearly up to ~1.5 fold as the amount of DNA-Cy3 doped in Alq_3_ particles increases, indicating that larger degree of doping enhances bio-recognitive photonics. Specific detection of DNA-doped Alq_3_ microparticles is also confirmed (Supplementary Fig. 11). Only complementary recognition triggers PL enhancement in the Alq_3_ particles. We then measured the optical waveguide characteristics of single isolated Alq_3_ particles with DNAs (T_20_, A_20_, G_20_, and C_20_) using a house-built laser confocal microscope (LCM) system with a diode laser (*λ*_ex_ = 405 nm). PL intensities of all Alq_3_ particles are decreased along the increasing propagation distance (Supplementary Fig. 12). In particular, the PL intensities have low optical transmission loss for Alq_3_ doped with DNAs (T_20_ and A_20_) (Fig. 4e). The optical loss coefficient of each Alq_3_ microparticle was calculated by exponentially fitting the emission intensities with respect to the propagation distance to distinguish the optical waveguide efficiency more quantitatively. Loss coefficient values, measured for the Alq_3_ particles doped with T_20_ (0.03701 μm^−1^) and A_20_ (0.04421 μm^−1^), are twice lower than those of the other Alq_3_ particles (Fig. 4f). However, compared to fluorescent enhancement, optical waveguide phenomena reveal similar loss coefficient values for T_20_ and A_20_ and G_20_ and C_20_. For the optical waveguide phenomena, light propagation is mainly affected by the surface of the Alq_3_ crystal^16^, whereas PL enhancement after treatment with complementary DNAs originates from the surface and inner core of the Alq_3_ particles. The molecular distribution of doped DNA with different base moieties at the Alq_3_ surface is shown in Supplementary Fig. 13. The fluorescence of the DNA (T_20_ and A_20_)-Cy3 molecules exhibits similar distribution, most intense in the central region along the longitudinal axis of the Alq_3_ surface. In contrast, DNA (G_20_ and C_20_)-Cy3 molecules demonstrate a lower intensity over the entire Alq_3_ surface. Hence, we deduce that a larger doping extent of DNA molecules at the Alq_3_ particle’s surface reduces the optical loss during light propagation in T_20_ and A_20_. Thus, the extent of DNA doping can tailor the biophotonic properties, including the bio-specific photonics and optical waveguide phenomenon, of light-emitting organic crystals.

## Discussion

In summary, we proposed a molecular-level interpretation of the doping behavior of DNAs in light-emitting organic particles by varying types and numbers of purine and pyrimidine bases constituting oligonucleotide. Intermolecular interactions with Alq_3_ particles, which induce molecular doping, originate from three noncovalent interactions: electrostatic, hydrogen bonding, and π-π stacking interactions. For the DNA length effect, stronger electrostatic interactions between the phosphate backbone of nucleic acid and Alq_3_ for longer nucleic acid molecules induce a higher degree of doping. For the bases effect, adenine and thymine molecules generate stronger attraction with Alq_3_ molecules due to the multisite interactions combining π-π stacking and hydrogen bonding interactions, in spite of low occurrence probabilities. This strong attraction contributes to a higher degree of doping than cytosine and guanine, which only form π-π stacking interactions with Alq_3_ molecules. In particular, MD simulations revealed that thymine molecules exert the strongest attraction through multisite interactions with single Alq_3_ molecules, facilitating the molecular doping into Alq_3_ particles up to 1.5-fold.

This molecular doping of nucleic acid molecules successfully tailors the resulting biophotonics of hybrid Alq_3_ particles in terms of (i) enhancing the recognitive photonics for complementary DNA molecules and (ii) reducing optical loss during the optical waveguide phenomena. We expect that rational designs of doped nucleic acids based on the fundamental understanding of intermolecular interactions can impact the technological applications of such DNA-hybrid light-emitting materials with tailored biophotonics.

## Methods

### Fabrication of DNA-doped Alq_3_ microparticles

A facile reprecipitation method for fabricating DNA-doped Alq_3_ microparticles has been previously reported^15,28^. Commercial Alq_3_ powder and tetrahydrofuran (≥99%) were purchased from Sigma–Aldrich. The oligonucleotides and oligonucleotides labeled with Cy3 fluorescent dyes used for doping were synthesized and purified by Bioneer Corporation (Daejeon, Korea). The quality of the synthesized oligonucleotides and quantity of labeled Cy3 molecules was verified using the MALDI-TOF mass spectrometer. The Alq_3_ powder was dissolved in tetrahydrofuran at a concentration of 1 mg mL^‒1^ to form a stock solution. The stock solution (2 mL) was injected into a series of 20 mL aqueous DNA solutions at a concentration of 0.8 μM with vigorous stirring (~800 rpm) for 2 min. The mixture was stored overnight at 25 °C to form a visible precipitate. The DNA sequences used in this study are listed in Supplementary Table 1. DNA was labeled with cyanine dye (Cy3) derivatives for molecular distribution and energy transfer analysis.

### Hybridization of DNA molecules doped into Alq_3_ microparticles

A method for hybridizing DNA-doped Alq_3_ microparticles with tDNA has been previously reported^15^. The DNA-doped Alq_3_ microparticles with different sequences (T_20_, A_20_, G_20_, or C_20_) were reacted with 0.8 μM complementary tDNA(A_20_, T_20_, C_20_, or G_20_) at 52 °C for 30 min and then cooled to 25 °C.

### Characterization of Alq_3_ microparticles

The morphology of the Alq_3_ particles was analyzed by field-emission SEM (Hitachi, S-4300) at an acceleration voltage of 15 kV. A holotomography microscope (HT-2H, Tomocube Inc., Republic of Korea)^39^ was performed to visualize 3D RI distributions of DNA doped Alq_3_ particles. The acquired images were processed via Tomostudio software (Tomocube Inc.). CLSM (Carl Zeiss, LSM700) provides z-sectioning fluorescence images of the Alq_3_ particles and DNA molecules. The Alq_3_ molecules were excited at 405 nm, and fluorescence was detected after passing through a 300‒550 nm filter. The DNA molecules labeled with Cy3 dye were excited at 550 nm, and fluorescence was detected after passing through a 300‒630 nm filter. The Alq_3_ particles were analyzed using a z-stack of images collected at 100 nm intervals. PL spectra were acquired using a fluorescence spectrophotometer (Hitachi, F-7000; excited by a xenon lamp). FRET efficiency between Alq_3_ particles and Cy3 fluorescence dyes was calculated by comparing the fluorescence intensities of the donor (Alq_3_) at 512 nm in the presence and absence of the acceptor (Cy3). The emission ratio (*Α*_572 nm_/*Α*_512 nm_) was calculated after the deconvolution of PL spectra of Alq_3_ particles by three gaussian peaks. X-ray diffraction (XRD; Rigaku, SmartLab) patterns were captured at a voltage of 40 kV and current of 40 mA using Cu-Kα radiation (*λ* = 1.540 Å). The optical waveguides of the Alq_3_ particles were measured using an in-build LCM system comprising a diode laser (*λ*_ex_ = 405 nm) for excitation and a spectrometer (Andor, SR-3031-B) for recording the PL spectra. Three to five Alq_3_ particles from each sample were used for calculating intensities of waveguided PL. We separated the energy irradiation and signal detection points using a piezoelectric stage (Nanofocus Inc., Albatross) to obtain precise waveguide intensity at each propagation point.

### AA MD simulations of DNA molecules on Alq_3_ particles

The all atomic simulation of DNA molecules on the Alq_3_ particles was performed by GROMACS 5.1.4, using the CHARMM36 force-field^40,41^. The simulations were conducted using a leapfrog integrator with a time step of 2 fs. PME with a cutoff of 1.2 nm calculated the electrostatic interactions^42^. Neighbor lists built using the Verlet cutoff scheme with a cutoff range of 1.2 nm were updated at every simulation step. The LINCS algorithm constrains the bond lengths^43^. All systems were maintained at 298.15 K using a V-rescale thermostat^44^ and at 1 bar using the Berendsen^45^ and Parrinello-Rahman^46^ barostats for the equilibrium and production runs, respectively.

The system consists of DNA[(GT)_x_G] (*x* = 2, 7, and 13) or 20-mer DNA molecules with different sequences (T_20_, A_20_, G_20_, or C_20_), 100 Alq_3_ molecules, and 30,000 pre-equilibrated water molecules in a simulation box with dimensions of 10 × 10 × 10 nm. The Alq_3_ molecule was parameterized based on the parameters developed by Voorhis et al.^47^. The interaction energies, including electrostatic energy and hydrogen bonding, between the DNAs and Alq_3_ particles, were calculated using the GROMACS analysis tool^40^. For the π-π stacking structures, the vertical separation between the base and quinoline was less than 0.45 nm^48^.

### AA MD simulation calculating the binding free energy of DNA molecules on Alq_3_ particles

Umbrella sampling simulations were conducted for each DNA to compute the binding energies of the DNA molecules. To construct initial configurations for binding free energy, we manually constrained the position of the backbone of DNA (A_20_), fully adsorbed on the Alq_3_ particle, and replaced adenine with other bases. Thereafter, the four systems with DNAs (T_20_, A_20_, G_20_, or C_20_) were equilibrated for 100 ns. To generate the z-directional reaction coordinate, DNA was pulled from the Alq_3_ particles using a force constant of 1000 kJ mol^‒1^ nm^‒2^. Forty windows with a spacing of 0.15 nm were created in each system. Equilibration and production runs of 1 and 10 ns, respectively, were performed for each window. WHAM tools in the GROMACS package measured the mean force potential. The binding energy is the difference between the highest and lowest energy states of the potential of mean force (PMF) curves (Supplementary Fig. 14).

## Supplementary information

Supplementary Information

## Data Availability

All data needed to evaluate the conclusions in this study are present within the article and Supplementary Information. Source data for main figure and Supplementary Information are provided with this paper. Additional data related to this paper are available from the corresponding author upon request. Source data are provided with this paper.

## Source data

Source Data
