# Supplementary material for: Molecular doping of nucleic acids into light emitting crystals driven by multisite-intermolecular interaction
Source: Nat Commun. 2022 Oct 19;13:6193. doi: 10.1038/s41467-022-33999-y (PMC9581973; doi:10.1038/s41467-022-33999-y)
Supplement: Supplementary file 1 — Supplementary Information [file 41467_2022_33999_MOESM1_ESM.pdf]

Molecular Doping of Nucleic Acids into Light Emitting Crystals Driven by Multisite-Intermolecular Interaction

Woo Hyuk Jung[1†], Jin Hyuk Park[1,2†], Seokho Kim[1†], Chunzhi Cui[1,3] & Dong June Ahn[1,2*]

[1]Department of Chemical and Biological Engineering, Korea University, Seoul 02841, Korea

[2]KU-KIST Graduate School of Converging Science and Technology, Korea University, Seoul 02841, Korea

[3]Department of Chemistry, National Demonstration Centre for Experimental Chemistry Education, Yanbian University, Yanji 133002, China

[*]*Correspondence and requests for materials should be addressed to D.J.A. (email: ahn@korea.ac.kr)*

This file contains following information:

Supplemental Figure 1: FRET effects of DNA-Cy3 doped in Alq_3 microparticles.

Supplemental Figure 2: SEM images of DNA-doped Alq_3 microparticles depending on DNA lengths.

Supplemental Figure 3: Fluorescent analysis of DNA-Cy3 doped Alq_3 microparticles fabricated with various concentrations of $(GT)_2G$-Cy3.

Supplemental Figure 4: DNA-doped Alq_3 microparticles fabricated under a variety of pH solution.

Supplemental Figure 5: Optical fluorescence properties of DNA-doped Alq_3 microparticles fabricated under a variety of pH solution.

Supplemental Figure 6: Morphological analysis of DNA-doped Alq_3 microparticles fabricated with different 20 mer-DNA bases.

Supplemental Figure 7: Fluorescent analysis of DNA-doped Alq_3 microparticles fabricated with different 20 mer-DNA bases.

Supplemental Figure 8: MD simulations for assembly of Alq_3 particles with different 20 mer-DNA bases.

Supplemental Figure 9: MD simulations for calculating adsorption energy of different 20 mer-DNA bases on Alq_3 crystal surface.

Supplemental Figure 10: Fluorescent analysis of DNA-doped Alq_3 microparticles with different bases before and after treatment with complementary DNAs.

Supplemental Figure 11: Specific Detection of DNA-doped Alq_3 microparticles fabricated with poly(T_{20}) DNA bases.

Supplemental Figure 12: Optical waveguide effects of Alq_3 microparticles with different bases after treatment with complementary DNAs.

Supplemental Figure 13: Surface molecular distribution of 20 mer-DNA with different base unities at the top plane of single Alq_3 particle.

Supplemental Figure 14: Potential of mean force curve for calculating the binding energy between DNA and Alq_3 particle.

Supplemental Table 1: Single-strand DNA and target DNA sequences used.

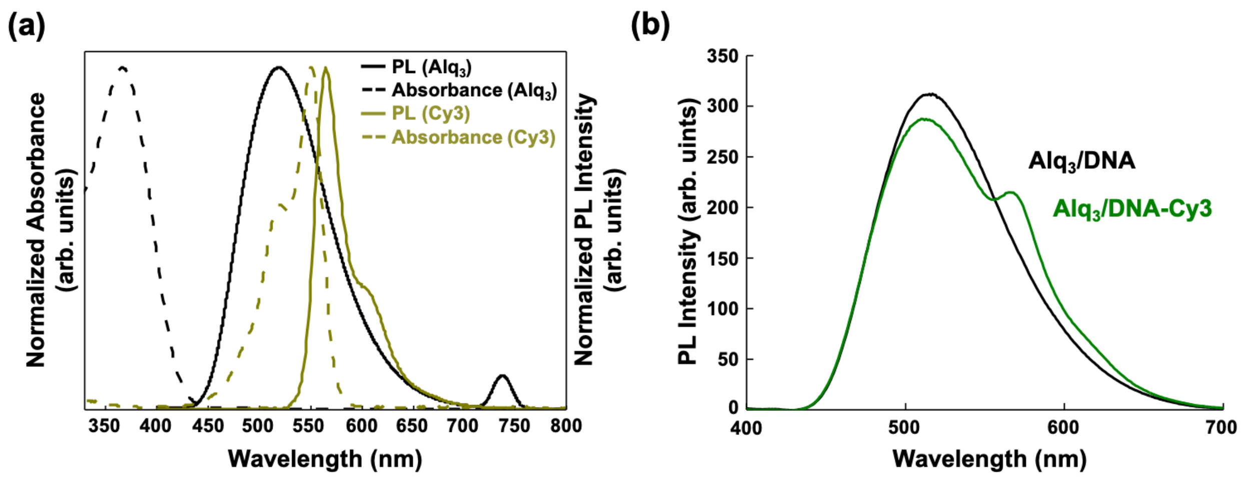

Supplementary Fig. 1 | FRET effects of DNA-Cy3 doped in Alq_3 microparticles. (a) Normalized optical absorption spectra (dash lines) and normalized PL emission spectra (solid lines) of DNA-Cy3 molecules (yellowish-green) and Alq_3 particles (black). (b) PL spectra of DNA doped Alq_3 microparticles (black) and DNA-Cy3 doped Alq_3 microparticles (green) averaged from eight separate experiments. The FRET efficiency was calculated based on the difference between the donor (Alq_3) fluorescence intensity in the presence and absence of the acceptor (Cy3).

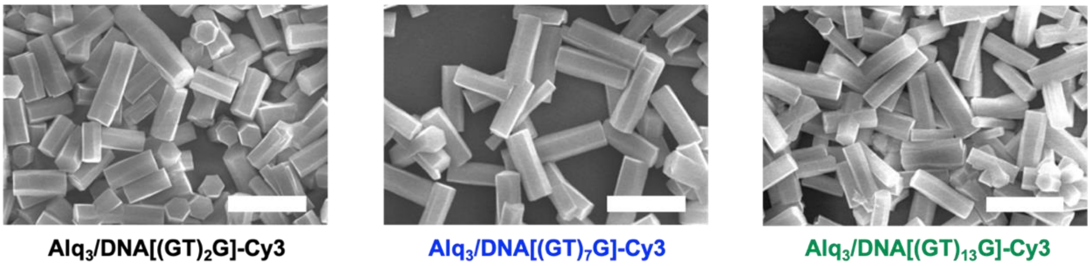

Supplementary Fig. 2 | SEM images of DNA-doped Alq_3 microparticles depending on DNA lengths. SEM images captured the Alq_3/DNA[$(GT)_x$G]-Cy3 particles x = 2, 7, and 13 (scale bar, 10 μm). The morphological size of particles is independent of the length of DNA doped in Alq_3 microparticles.

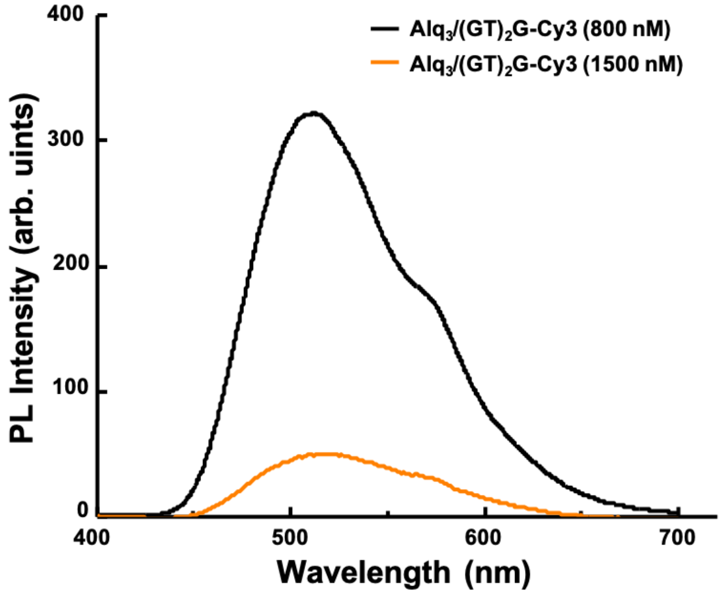

Supplementary Fig. 3 | Fluorescent analysis of DNA-Cy3 doped Alq_3 microparticles fabricated with various concentrations of $(GT)_2G$-Cy3. PL spectra of Alq_3 microparticles fabricated with 800 nM $(GT)_2G$-Cy3, and 1500 nM $(GT)_2G$-Cy3. The emission ratio ($A_{572\ nm}/A_{512\ nm}$) was calculated by comparing the Gaussian peak area at 572 nm with the Gaussian peak area at 512 nm after the deconvolution.

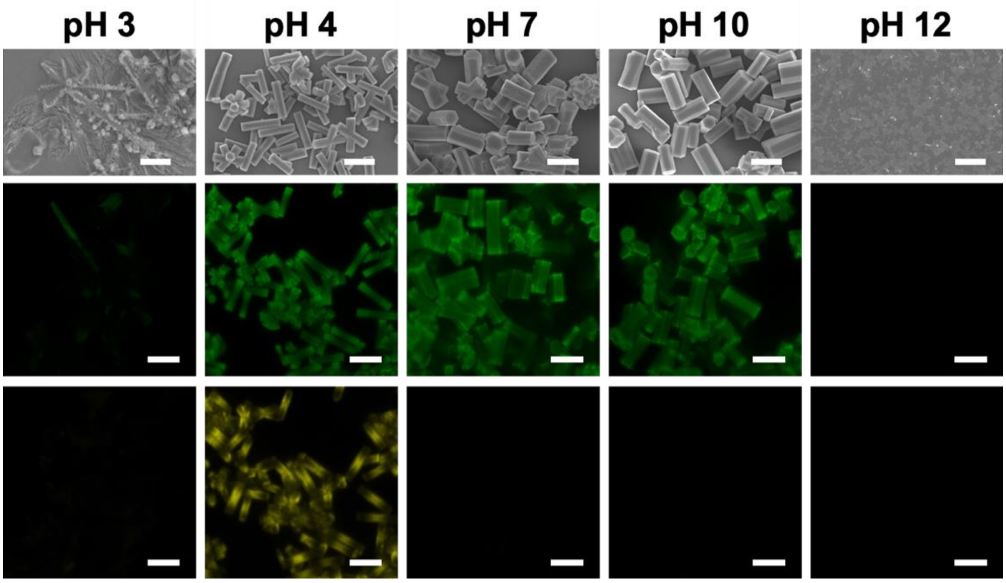

Supplementary Fig. 4 | DNA-doped Alq_3 microparticles fabricated under a variety of pH solution. SEM images (top), CLSM images of Alq_3 (middle) and DNA-Cy3 molecules (bottom) captured at the center plane of DNA-doped Alq_3 microparticles fabricated at pH 3, 4, 7, 10, and 12, respectively (scale bar, 10 μm). The images of the Alq_3 molecules are obtained by excitation using a 405 nm laser; a 300-550 nm filter is used. The images of the DNA-Cy3 molecules are obtained by excitation with a 555 nm laser; a 300–630 nm filter is used. The DNA sequence used for fabrication of the particles is 5`-ATC CTT ATC AAT ATT TAA CAA TAA TCC-3`.

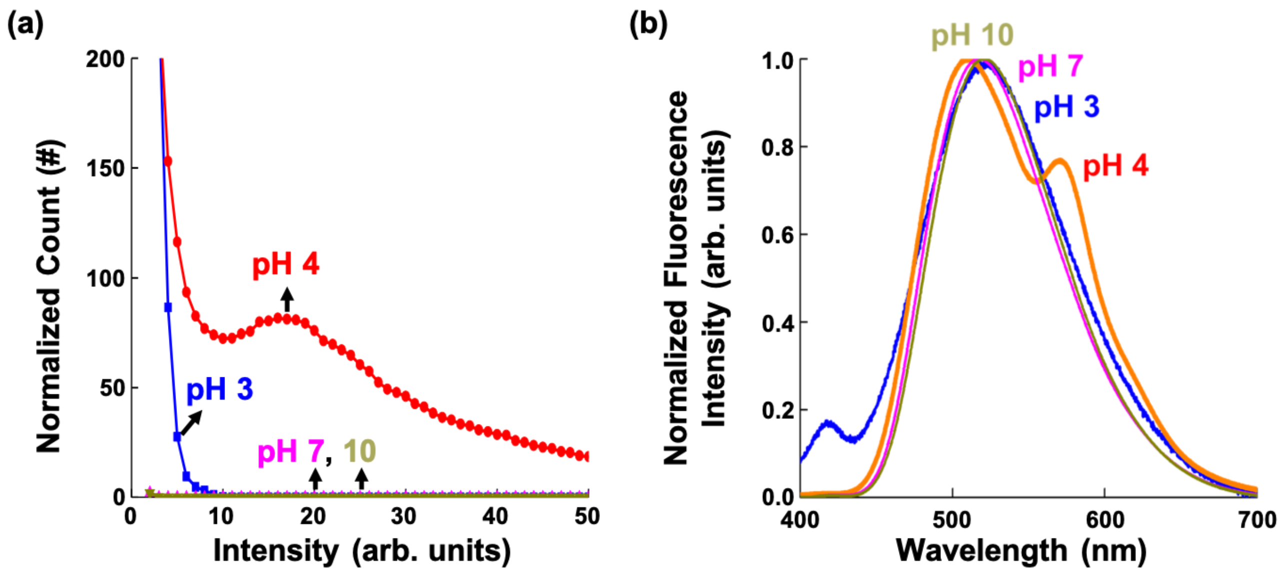

Supplementary Fig. 5 | Optical fluorescence properties of DNA-doped Alq_3 microparticles in Supplementary Fig. 4. (**a**) Quantitative fluorescence histogram of DNA-Cy3 molecules doped in Alq_3 particles, and (**b**) photoluminescence (PL) spectra of DNA-Cy3-Alq_3 microparticles, fabricated at different pH, with excitation at 365 nm. Particles were not fabricated at pH 12.

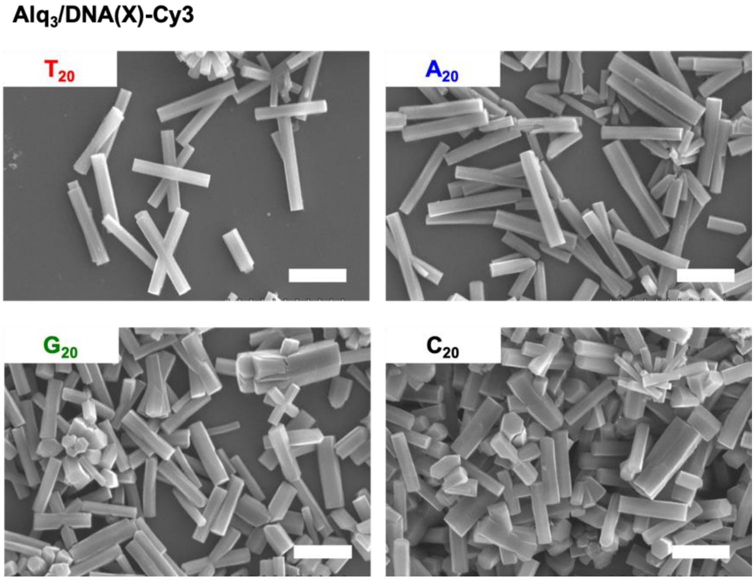

Supplementary Fig. 6 | Morphological analysis of DNA-doped Alq_3 microparticles fabricated with different 20 mer-DNA bases. SEM images of Alq_3/DNA(T_{20}, A_{20}, G_{20}, and C_{20}) (scale bar, 10 μm).

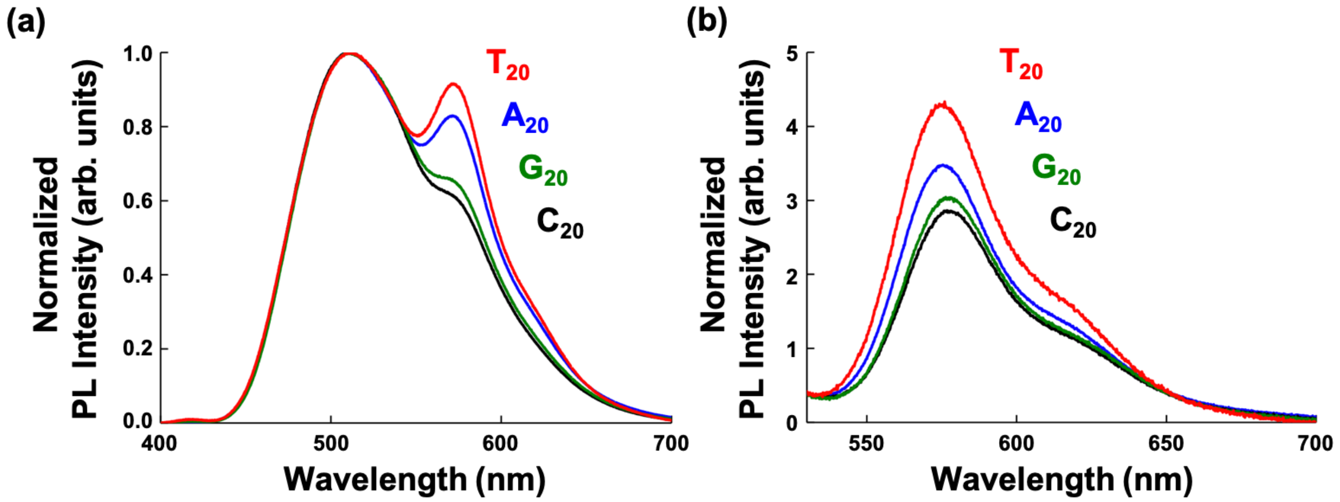

Supplementary Fig. 7 | Fluorescent analysis of DNA-doped Alq_3 microparticles fabricated with different 20 mer-DNA bases. PL spectra of Alq_3/DNA(A_{20}, C_{20}, G_{20}, and T_{20})-Cy3 with (a) excitation at 365 nm showing FRET properties and (b) 500 nm indicating DNA-Cy3 molecules in the particles.

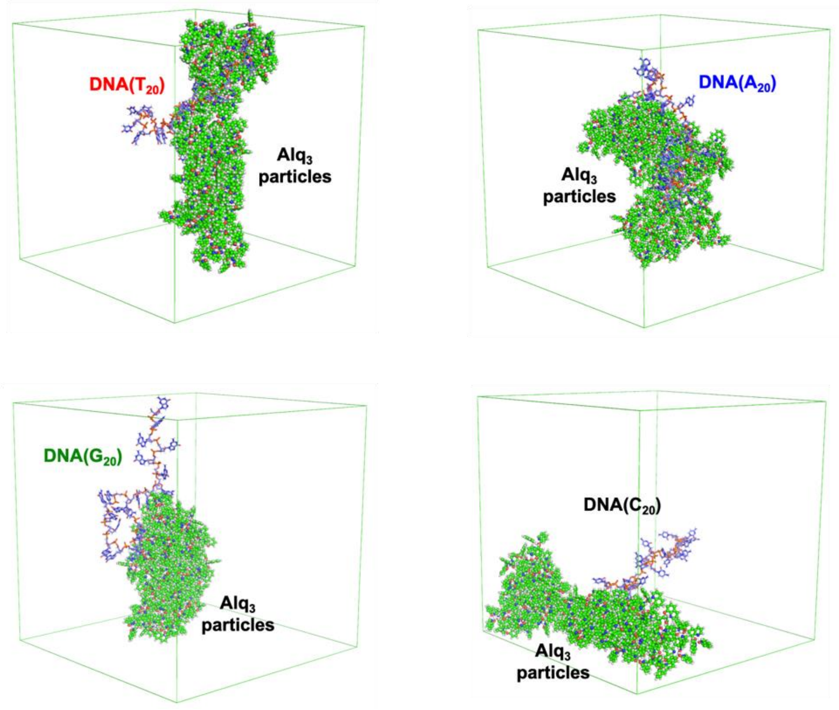

Supplementary Fig. 8 | MD simulations for assembly of Alq_3 particles with different 20-mer DNA bases. Final configurations of Alq_3 particles assembled with DNA (T_{20}, A_{20}, G_{20}, and C_{20}) after 200 ns simulations. The initial systems were constructed with 100 Alq_3 molecules and single DNA segment with different bases.

(a)

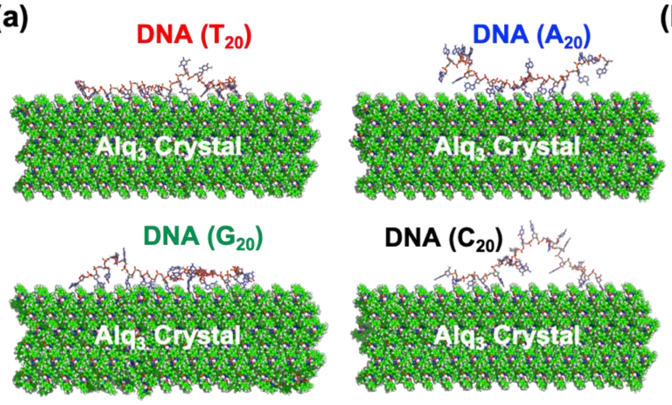

(b)

	Adsorption Energy (kJ/mol)
Alq_3+DNA(T_{20})	-6007.4 ± 266.6
Alq_3+DNA(A_{20})	-5606.5 ± 291.2
Alq_3+DNA(G_{20})	-5732.2 ± 261.6
Alq_3+DNA(C_{20})	-5796.3 ± 115.2

Supplementary Fig. 9 | MD simulations for calculating adsorption energy of different 20 mer-DNA bases on Alq_3 crystal surface. (a) Final configurations of DNA (T_{20}, A_{20}, G_{20}, and C_{20}) on Alq_3 crystal surface and (b) their corresponding adsorption energy.

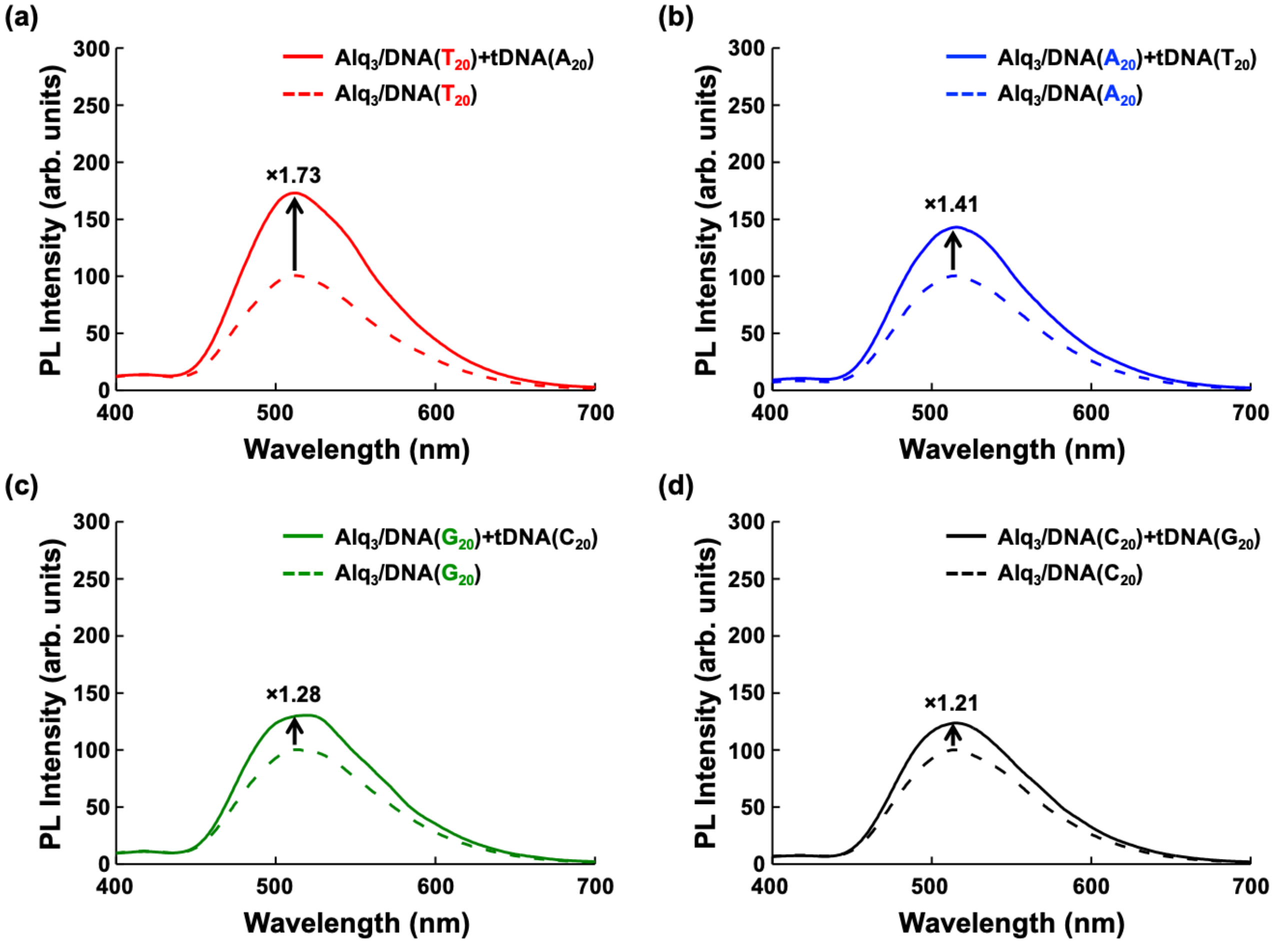

Supplementary Fig. 10 | Fluorescent analysis of DNA-doped Alq_3 microparticles with different bases before and after treatment with complementary DNAs. (a-d) PL spectra of Alq_3/DNAs (T_{20}, A_{20}, G_{20}, and C_{20}) before and after treatment with corresponding complementary DNAs (excitation at 365 nm). The ratio of PL enhancement after treatment with complementary DNAs is calculated based on the intensity of main PL peaks of Alq_3 particles (512 nm).

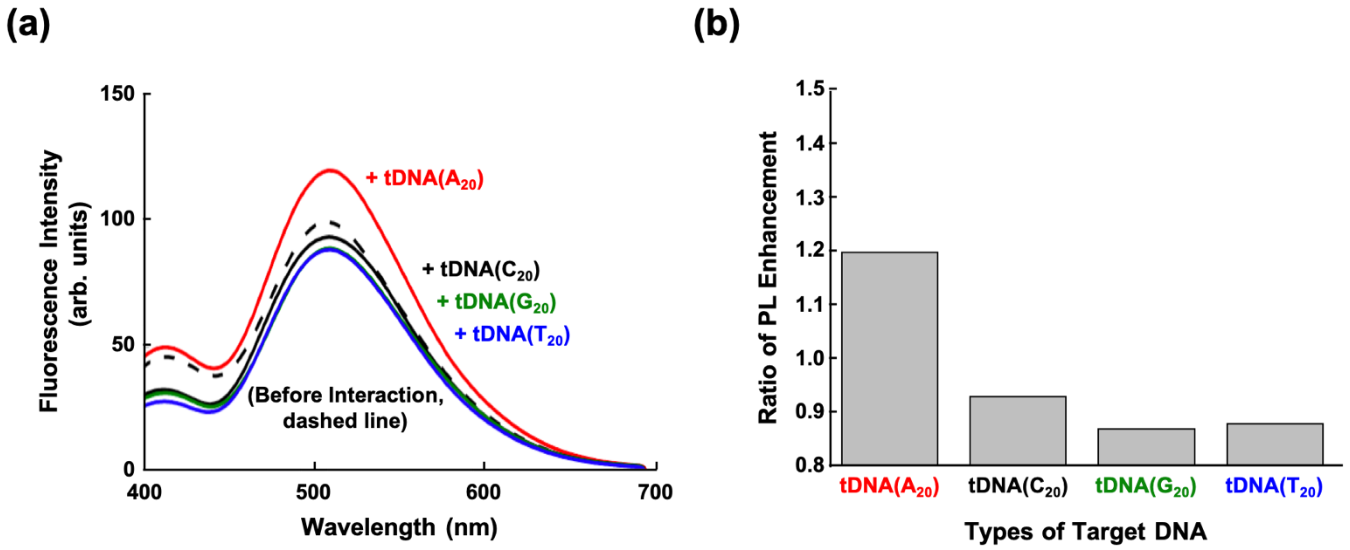

Supplementary Fig. 11 | Specific Detection of DNA-doped Alq_3 microparticles fabricated with poly(T_{20}) DNA bases. (a) PL spectra with excitation at 365 nm and (b) PL enhancement ratio of DNA(T_{20})-doped Alq_3 microparticles after treatment with various target DNAs (A_{20}, C_{20}, G_{20}, and T_{20}) at 8 nM. Only complementary DNA(T_{20})-tDNA(A_{20}) recognition triggers PL enhancement of Alq_3 particles.

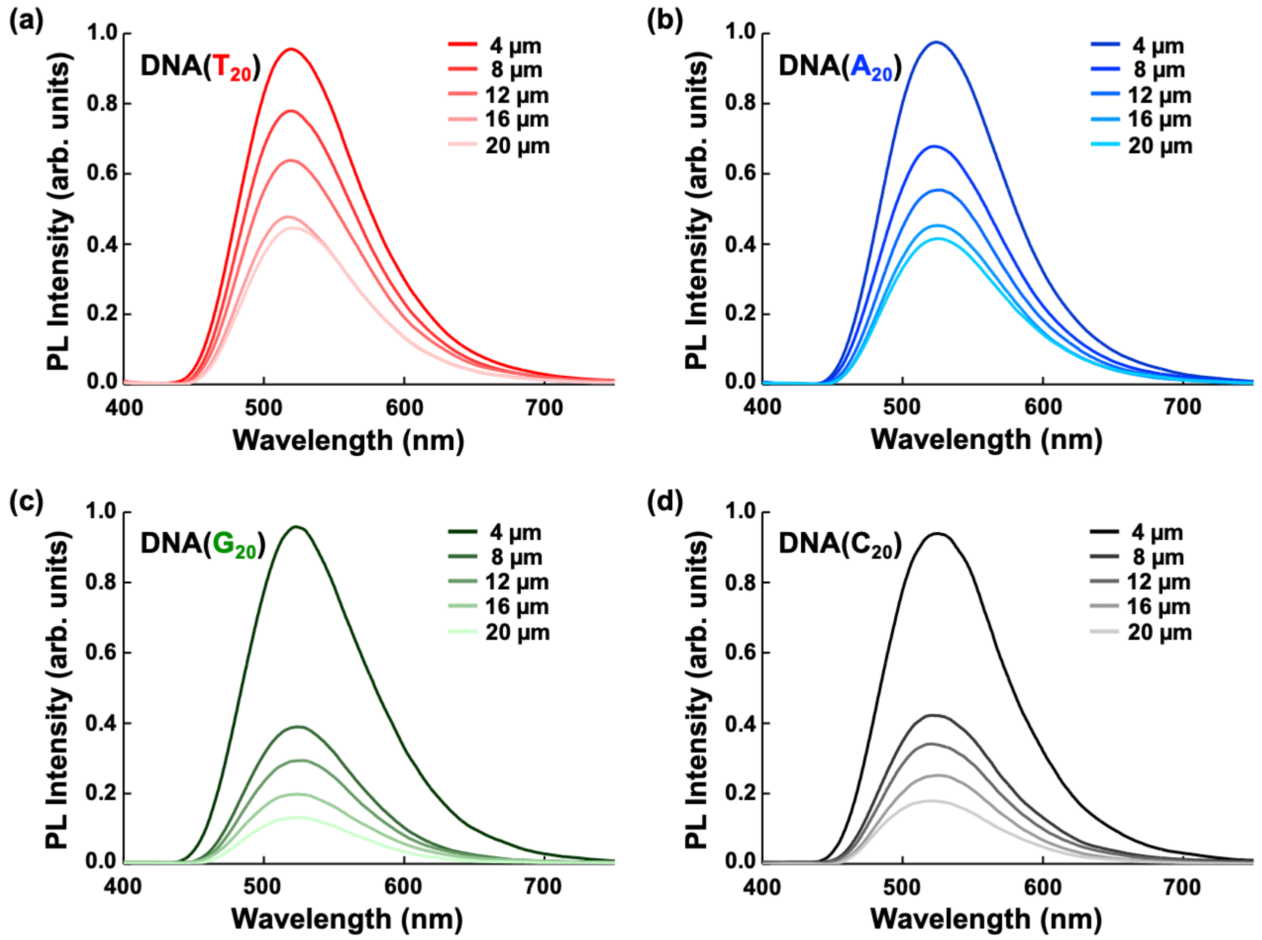

Supplementary Fig. 12 | Optical waveguide effects of Alq_3 microparticles with different bases after treatment with complementary DNAs. (a-d) Comparison of waveguide PL intensities of Alq_3/DNAs (T_{20}, A_{20}, G_{20}, and C_{20}) microparticles after treatment with corresponding complementary DNAs according to the propagation distance.

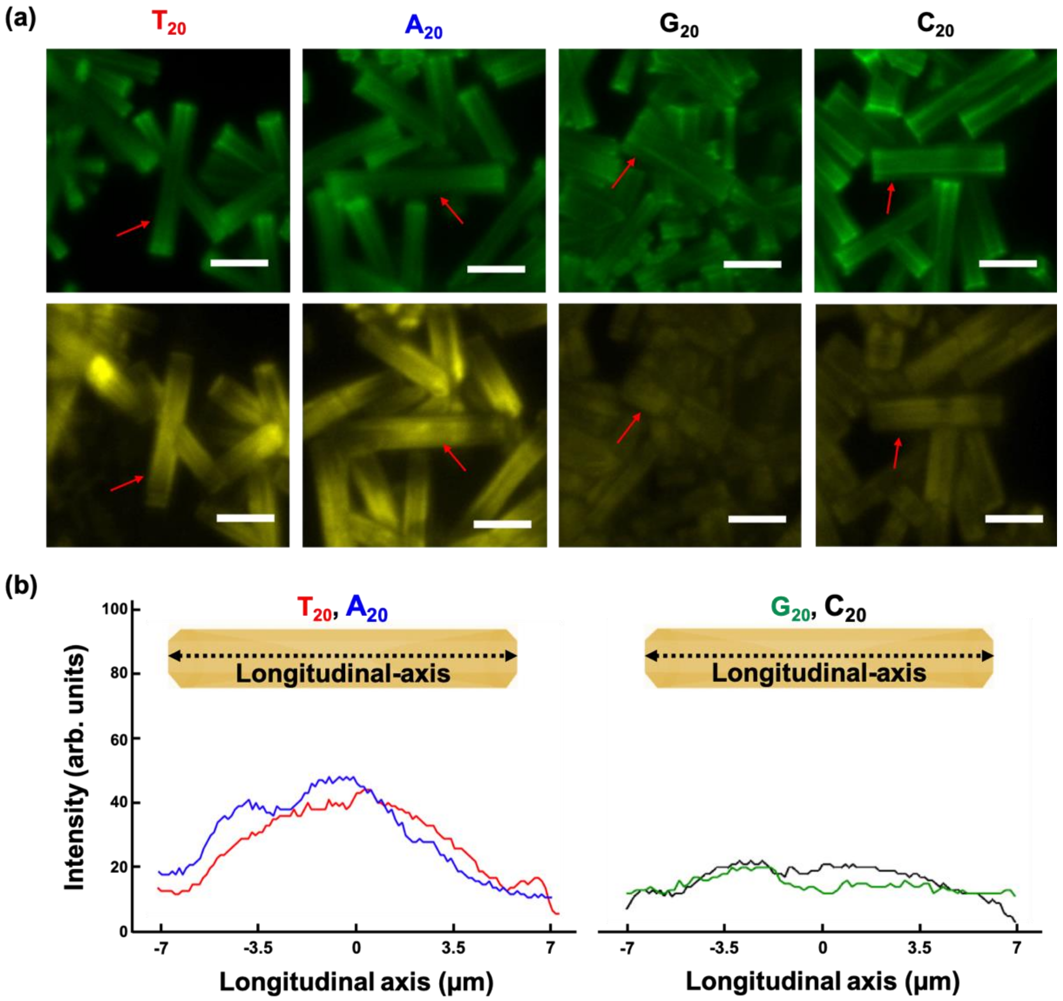

Supplementary Fig. 13 | Surface molecular distribution of 20 mer-DNA with different base unities at the top plane of single Alq_3 particle. (a) CLSM images captured at the upper plane of Alq_3/DNA(A_{20}, C_{20}, G_{20}, and T_{20})-Cy3 particles (scale bar, 5 μm). The top panels are obtained with excitation of the molecules using a 405 nm laser; a 300-550 nm filter is used to capture Alq_3 particles. The bottom panels are obtained by excitation with a 555 nm laser; a 300-630 nm filter is used to capture DNA-Cy3 molecules. (b) Longitudinal fluorescence profiles quantifying local intensity of DNA(A_{20} and T_{20})-Cy3 molecules (left) and DNA(C_{20} and G_{20})-Cy3 molecules (right) at the upper plane of single Alq_3 particle.

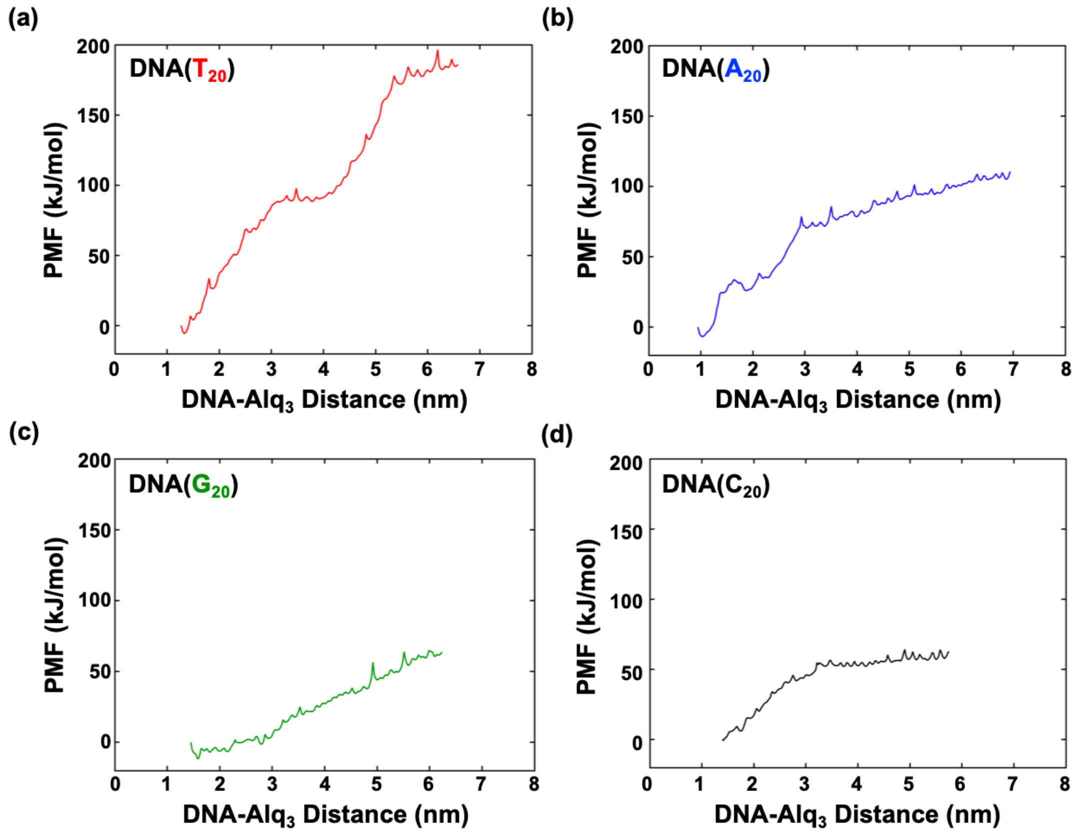

Supplementary Fig. 14 | Potential of mean force curve for calculating the binding energy between DNA and Alq$_3$ particle. (a-d) Potentials of mean force curve of one replicate as functions of the distance between centers of mass of DNAs (T_{20}, A_{20}, G_{20}, C_{20}) and Alq$_3$ particle. The umbrella sampling of DNAs was terminated after the detachment of DNAs from Alq$_3$ particles, which were confirmed when the separation between atoms of quinolines and bases was larger than 0.52 nm.

Supplementary Table 1 | Single-strand DNA and target DNA sequences used.

**Abbreviation**	**DNA Sequence**
DNA (5 mer)-Cy3[a]	5`-GTGTG-3`-Cy3
DNA (15 mer)-Cy3	5`-GTGTGTGTGTGTGTG-3`-Cy3
DNA (27 mer)-Cy3	5`-GTGTGTGTGTGTGTGTGTGTGTGTGTG-3`-Cy3
anthrax DNA-Cy3[b]	5`-ATCCTTATCAATATTTAACAATAATCC-3`-Cy3
anthrax tDNA	3`-TAGGAATAGTTATAAATTGTTATTAGG-5`
DNA(A_{20})-Cy3	5`-AAAAAAAAAAAAAAAAAAAA-3`-Cy3
DNA(C_{20})-Cy3	5`-CCCCCCCCCCCCCCCCCCCC-3`-Cy3
DNA(G_{20})-Cy3	5`-GGGGGGGGGGGGGGGGGGGG-3`-Cy3
DNA(T_{20})-Cy3	5`-TTTTTTTTTTTTTTTTTTTT-3`-Cy3
tDNA(T_{20})	3`- TTTTTTTTTTTTTTTTTTTT -5`
tDNA(G_{20})	3`- GGGGGGGGGGGGGGGGGGGG -5`
tDNA(C_{20})	3`- CCCCCCCCCCCCCCCCCCCC -5`
tDNA(A_{20})	3`- AAAAAAAAAAAAAAAAAAAA -5`

[a] Cy3= cyanine 3 dye. [b] Anthrax DNA is anthrax lethal factor probe single stranded DNA sequence.
